# The Swedish Renal Registry: a nationwide registry for chronic kidney disease of all stages

**DOI:** 10.1093/ckj/sfaf238

**Published:** 2025-08-06

**Authors:** Anne-Laure Faucon, Helena Rydell, Maria Stendahl, Gunilla Welander, Torbjörn Lundgren, Karl-Göran Prûtz, Staffan Schön, Ursa Bonnevier, Aline Kåveryd Hult, Håkan Hedman, Frida Fondelius, Mårten Segelmark, Marie Evans

**Affiliations:** Department of Medical Epidemiology and Biostatistics, Karolinska Institutet, Stockholm, Sweden; Department of Clinical Epidemiology, Centre for Research in Epidemiology and Population Health, INSERM U1018, Paris-Saclay University, France; Department of Nephrology, Karolinska University Hospital, Stockholm, Sweden; Department of Clinical Science, Intervention and Technology, Karolinska Institutet, Stockholm, Sweden; Department of Medicine, Ryhov Hospital, Region Jönköping, Sweden; Department of Nephrology, Karlstad Hospital, Karlstad, Sweden; Department of Medical Sciences, Uppsala University, Uppsala, Sweden; Department of Clinical Science, Intervention and Technology, Karolinska Institutet, Stockholm, Sweden; Department of Transplant Surgery, Karolinska University Hospital, Stockholm, Sweden; Swedish Renal Registry, Region Jönköping, Jönköping, Sweden; Swedish Renal Registry, Region Jönköping, Jönköping, Sweden; Department of Medical Sciences, Uppsala University, Uppsala, Sweden; Department of Nephrology, Gävle Hospital, Gävle, Sweden; Transplant Centre, Sahlgrenska University Hospital, Göteborg, Sweden; Swedish Renal Registry, Region Jönköping, Jönköping, Sweden; Swedish Renal Association, Stockholm, Sweden; Department of Endocrinology, Nephrology and Rheumatology, Skåne University Hospital, Lund, Sweden; Department of Endocrinology, Nephrology and Rheumatology, Skåne University Hospital, Lund, Sweden; Department of Clinical Sciences, Lund University, Lund, Sweden; Department of Nephrology, Karolinska University Hospital, Stockholm, Sweden; Department of Clinical Science, Intervention and Technology, Karolinska Institutet, Stockholm, Sweden

**Keywords:** chronic kidney disease, clinical practices, dialysis, registry, transplantation

## Abstract

**Background:**

National registries that capture patients with chronic kidney disease (CKD) across all stages are scarce. We present here the Swedish Renal Registry (SRR), a nationwide prospective register covering the whole spectrum of CKD.

**Methods:**

Created in 1991, SRR enrolls CKD patients referred to adult nephrologist care (non-dialysis CKD [ND-CKD], kidney transplantation, maintenance dialysis), with an overall coverage of nearly all nephrology clinics in Sweden. SRR encompasses several interconnected databases in which longitudinal clinical, biological, kidney-related, patient-reported data, as well as provider-level information are collected. This report presents the design of the registry, as well as patients characteristics and temporal trends between 2008 and 2021.

**Results:**

A total of 45 590 new ND-CKD patients [72 years, 16 656 (36%) women, eGFR 26 ml/min per 1.73 m²], and 8829 incident kidney transplant recipients (51 years, 35% women) were enrolled in SRR between 2008 and 2021. SRR also included 16 034 new patients [68 years, 5420 (34%) women] on maintenance dialysis [70% hemodialysis (HD), 30% peritoneal dialysis (PD)]. Between 2015 and 2021, 4753 patients [59 years, 1884 (40%) women, eGFR 37 ml/min per 1.73 m²] had a registered kidney biopsy. We observed a decrease in HD incidence (69% to 60%, *P* for trend <.01) and an increase in PD incidence (26% to 32%, *P* < .01) and pre-emptive transplantation (4.7% to 7.5%, *P* < .01). The prevalence of comorbidities is high in all CKD stages and increase as eGFR declines. While nephroangiosclerosis and diabetic kidney disease are the most common etiologies, glomerulonephritis are the most frequent diagnoses in biopsied and transplanted patients.

**Conclusion:**

The SRR is a nationwide register which aims to contribute to address gaps in our understanding of CKD, to identify important challenges and health priorities, evaluate real-life clinical management and analyze international variations, improve health outcomes, improve quality of life, and reduce the burden of CKD.

## Graphical Abstract

**Graphical Abstract:**
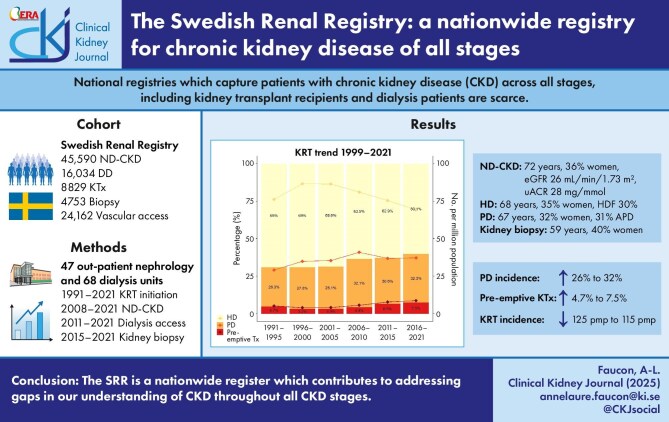

KEY LEARNING POINTS **What was known:**

- CKD is a common condition that affects 10%–15% of the adult population, and is associated with an excess risk of adverse outcomes.
- Although several clinical and epidemiological CKD cohorts exist, registries that capture individuals with CKD of all stages are scarce.

**This study adds:**

- The Swedish Renal Registry (SRR) is a nationwide and evolutive real-life register covering all stages of CKD.
- The SRR encompasses several interconnected databases, including data on non-dialysis CKD, hemodialysis and peritoneal dialysis, dialysis access, transition between dialysis modalities/transplant status, kidney biopsy, and patient-reported outcomes.
- Overall, patients with CKD have a very high-risk profile, characterized by old age, a high burden of comorbidities, polypharmacy, a poor quality of life, and a substantial risk of vascular access complications.

**Potential impact:**

- SRR aims to contribute to address gaps in our understanding of CKD, identify important challenges and health priorities, evaluate clinical management, and improve health outcomes.
- Linkages between SRR and several national Government-run registries provide extensive information on comorbidities, outcomes, medications, and vital status, among others, and could be used as an important real-life research platform.
- The SRR is a part of several international initiatives, such as via its collaboration with the European Renal Association (ERA) Registry, with the aim of identifying gaps in clinical practice and reducing the burden of CKD.

## INTRODUCTION

Chronic kidney disease (CKD) is a common condition that affects ~10%–15% of the adult population worldwide and is recognized as one of the ten most important non-communicable diseases of the 21st century [1, 2]. Overall incidence of CKD and kidney replacement therapy (KRT) steadily had increased over recent decades, mainly due to population aging and increased CKD risk factors such as diabetes and hypertension, and it is associated with substantial healthcare costs [3]. Kidney diseases consist of a broad range of clinically distinct etiologies, including kidney diseases due to cardio-metabolic disorders, systemic auto-immune diseases, infections, drug toxicity, hematologic/solid malignancies, obstructions, and genetic diseases. Regardless the underlying etiology, CKD is associated with a large set of complications, including a disproportionate high-risk of cardiovascular disease [4–9], metabolic complications and hydro-electrolytic disorders [10–15], kidney events [16, 17], and death [4, 5], and several conditions such as infections [18, 19], fractures [20], cancer [21], and cognitive impairment [22, 23], which are exacerbated as kidney function declines. CKD is also associated with a major reduction of quality of life (QoL) and life expectancy [24].

In Europe and other parts of the world, registries designed to follow patients with advanced CKD undergoing dialysis or kidney transplantation have existed for decades [25, 26]. A recent report from the European Renal Association (ERA) Registry shows that the ERA Registry currently covers data for patients with KRT from 53 registries in 35 different countries [26]. Patients initiating dialysis are relatively easy to identify and, because they are closely followed within nephrology care, it has been possible to systematically collect data on outcomes, such as mortality and transitions between treatment modalities (hemodialysis, PD, and kidney transplantation). As a result, research and knowledge about dialysis-related trajectories and outcomes have steadily grown, contributing to the development of evidence-based treatments. By contrast, there are few registries that collect data across the whole range of CKD—from the etiological diagnosis using kidney biopsy, to kidney disease progression in earlier CKD stages, to end-stage kidney disease, changes between dialysis modalities, and kidney transplantation. Such data are needed, for instance, to better understand determinants at the patient, physician, and healthcare system levels to reduce risks of starting dialysis in emergency and/or without any prior nephrology care [27, 28]; but also to analyze physical and psychosocial dimensions involved in transition to KRT to provide tailored care to ensure a smooth transition, because transition to KRT constitutes a critical period accompanied by life changes in patients who are often frail and vulnerable [29].

In Sweden, epidemiological research is facilitated thanks to the personal identification number unique for each citizen [30], which offers the possibility to link data from different sources, i.e. collected clinical data, healthcare quality registries [31], and Government-run national registries. The Swedish Renal Registry (SRR) was created in 1991 to inform patients and healthcare providers about the KRT burden in Sweden, and in 2008, it was expanded to include individual-level data on incident nephrology-referred patients of all CKD stages, including incident non-dialysis dependent CKD (CKD-ND) patients and incident patients starting KRT. Over the years, the SRR has contributed to research and nephrology care improvement through important insights about health trajectories, pathophysiological mechanisms, risk factors and biomarkers, prognosis, risk stratification, health outcomes, evaluation of clinical practices, and cost-effectiveness. Even though the SRR has contributed to substantial scientific advances since it was first created, there is no contemporary description of its construction and recent developments. In the present article, we provide a detailed description of the different interconnected parts of the SRR. We also present a description of the main patients’ characteristics and temporal trends between 2008 and 2021, thus providing data on a national level about the continuum of nephrology-referred CKD patients in a European country over the past 15 years.

## MATERIALS AND METHODS

### Swedish Renal Registry organization and ethical aspects

The Swedish healthcare system is based on a universal tax-funded access (both public and private providers) and divided into 21 independent regions (counties) throughout Sweden. The SRR is nationwide prospective quality register enrolling patients with CKD referred to adult nephrologist care, without any restriction of CKD stage, CKD etiology, age, ethnicity, or comorbidities, organized under Region Jönköping, Sweden (https://www.medscinet.net/snr/).

The SRR is one of many healthcare quality registers in Sweden, supervised by the National Board of Health and Welfare, and co-funded by the Swedish Association of local Authorities and Regions. The SRR currently employs two administrators and is run by a Steering committee consisting of 11 members: eight appointed by the Swedish Renal Association, one representative for the Swedish Renal Nurse Association, one for the Swedish Transplant Association, and one member representing the Patient Organization (Njurförbundet).

All patients are informed on entry to the SRR and have the right to opt out. According to the governing laws for healthcare quality registers in Sweden, no written informed consent is required. All research based on data from SRR must be approved by the national ethics committee, adhere to applicable General Data Protection Regulation (GDPR) regulations within the European Union, and be further approved by the legal authority in Region Jönköping.

### Swedish Renal Registry design

**Figure 1:**
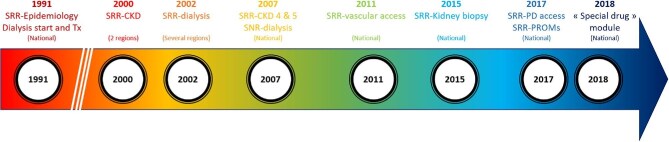
Timeline of the creation of the SRR.

A total of 47 clinics with non-dialysis CKD patients and 68 departments providing dialysis care participate in the SRR. SRR encompasses several national interconnected databases (Fig. 1, Table 1): SRR-epidemiology, SRR-CKD, SRR-kidney biopsy, SRR-dialysis, SRR-access, SRR-PROMs and QoL, and SRR-special drugs.

**Table 1:** List of variables collected in the SRR.

SRR-Epidemiology	
dates	date of KRT start modality transition
demographics^a^	date of birth, sex, clinic, county, region
comorbidities	etiology of nephropathy (2012-ERA coding system), cancer including skin cancer, diabetes, ischemic heart disease, heart failure, atrial fibrillation, hypertension, cerebrovascular disease, peripheral artery disease, smoking
KRT modality	HD, PD, Tx
kidney transplantation	date, no of Tx, type of donor
laboratory data	last creatinine value before KRT
SRR-CKD	
clinical data	SBP^a^, DBP^a^, weight, height, BMI
visit type^a^	date of visit, CKD/Tx
biological data	serum creatinine^a^, cystatin C, albumin^a^, uACR^a^, CRP^a^, Hb^a^, K, total Ca^a^, Phosphate^a^, PTH^a^, HbA1c, ferritin, transferrin, total cholesterol, HDL cholesterol, creatinine clearance, bicarbonates, triglycerides, TSAT, urea, uric acid, urine volume
diagnoses at baseline^a^	etiology of nephropathy (ERA coding system from 2012), cancer including skin cancer, diabetes, ischemic heart disease, heart failure, atrial fibrillation, hypertension, cerebrovascular disease, peripheral artery disease
medications	iron, iron type, ESA (name, week dose), phosphate binder, statin, vitamin D, calcimimetic, immunosuppressive therapy (po or iv), SGLT2 inhibitors, diuretic, number of antihypertensive drugs, name and class of antihypertensive medications
follow-up^a^	date of end of follow-up and reason, death and cause of death (ERA coding system)
patient education	date and type of education, modality choices and date (including conservative care)
SRR-dialysis	
modality^a^	hemodialysis: conventional HD, hemodiafiltration, hemofiltration PD: automated PD, continuous ambulatory PD
location of dialysis^a^	institution, home-HD, HD limited care, assisted PD
dates^a^	start of dialysis, visit date
dialysis access^a^	catheter, AV fistel/graft, PD catheter
dialysis prescription^a^	dialysis duration, weekly frequency, type of dialysate, PD volume
clinical data	SBP, DBP, weight, height (some clinical parameters are collected before and after dialysis session)
laboratory data	residual kidney function, Kt/v, serum creatinine^a^, cystatin C, albumin^a^, CRP^a^, Hb^a^, K, total Ca^a^, phosphate^a^, PTH^a^, HbA1c, ferritin, transferrin, total cholesterol, HDL cholesterol, creatinine clearance, bicarbonates, triglycerides, TSAT, urea, uric acid
medications	iron, iron type, ESA (name, week dose), phosphate binder, statin, vitamin D, calcimimetic, immunosuppressive therapy (per oral or intravenous), diuretic, number of antihypertensive drugs, name and class of antihypertensive medications
SRR-Dialysis access	
type of dialysis access	central dialysis catheter (tunneled or not), arteriovenous fistula/graft, PD catheter
location	upper arm/forearm, leg, side
dates	access creation/insertion, first puncture, function start, access loss
complications	thrombosis, stenosis, aneurysm, steal, bleeding, vascular access infection, high/low flow PD catheter dysfunction, leakage and other mechanical complications, peritonitis and other infections
interventions	thrombolysis, endovascular and surgical interventions, laparoscopic interventions for PD catheters
SRR-Kidney biopsy	
demographics	date of birth, sex
clinical data	SBP^a^, DBP^a^, weight, height, BMI, hereditary kidney disease, smoking, diagnosis of nephropathy (ERA coding system)
biopsy data	indication, location
laboratory data^a^	same as for SRR-CKD registry + hematuria, immunology data, serologies (hepatitis, HIV)
complications^a^	bleeding, transfusion, nephrectomy, readmission, death, intervention
pathohistological data	SNOMED codes, number of glomeruli, method (light microscopy, immunofluorescence/immunohistochemistry, electronic microscopy)
medications^a^	anticoagulants, antiplatelet, desmopressin, antihypertensive drugs
SRR-PROMs	
QoL	RAND36 (8 dimensions)
SRR-special drugs	
tolvaptan	indication, date of start/stop, dose changes, complications
HIF inhibitors	indication, date of start/stop, dose changes, complications
difelikefalin	indication, date of start/stop, dose changes, complications, pruritus score

a Mandatory variables in the registry. All data are collected at the date of the visit, except demographics and comorbidities where information is entered at inclusion.

CRP, C reactive protein; DBP, diastolic blood pressure; HDL, high-density lipid; HIF, hypoxia inducible factor; PTH, parathyroid hormone; SBP, systolic blood pressure; Tx, transplantation.

#### SRR-epidemiology

This section constitutes a pivotal component of the registry from which SRR has been developed, with the enrichment of all the other SRR databases. It was launched already in 1991, but also included retrospective data on kidney transplanted patients as far back as the 1960s. The SRR-epidemiology includes information on the starting date and modality of KRT, as well as information on transitions between dialysis modalities, and kidney transplant status. All patients starting dialysis are included, regardless of whether they survive 3 months or not, a common inclusion criterion in many other registries on chronic dialysis patients. All clinics providing KRT report to the SRR-epidemiology; the coverage is 100%. Validation analyses has estimated that >96% of kidney transplanted patients are included in the registry and almost all dialysis patients [32].

#### SRR-CKD

The collection of CKD-ND data started in 2000 in two regions (Gothenburg and Stockholm) and was developed to reach the national level in 2007. All non-dialysis patients with CKD, regardless of CKD stage, etiology, kidney transplanted or not, referred to nephrology care are eligible to be included in the registry, but it is only mandatory to include patients with an estimated glomerular filtration rate (eGFR) <30 ml/min/1.73 m². For clinics that chose to include patients earlier than CKD stage 4, they should be consistent and apply their inclusion criteria (for example eGFR <45 ml/min/1.73 m^2^) to all their patients. After registration, a minimum of one outpatient nephrology visit is collected every year (although many clinics register all visits), until death, start of KRT, or emigration from the country. SRR-CKD collects a large set of clinical and biological data—including CKD biomarkers of kidney function and kidney damage such as serum/plasma creatinine, cystatin C, urine albumin-to-creatinine ratio (uACR), allowing CKD staging according to the KDIGO categories—as well as selected comorbidities, medications, educational activities, and the vital status. SRR-CKD also collects the etiology of kidney disease assigned by the patient's nephrologist, and classified according to the European Renal Association for Primary Renal Disease (ERA-PRD) 2012 coding system [33]. The proportion of participating outpatient clinics is 98%, and validation analyses have demonstrated a coverage of 70%–96% (according to the different regions) of all patients with CKD stage 4–5 under nephrology care [34].

#### SRR-kidney biopsy

This was introduced in 2015. It includes renal pathology data from incident native kidneys. It collects biopsy indication such as “nephrotic syndrome,” “acute/subacute nephritic syndrome,” “other form of acute kidney injury,” “CKD stages 1–2,” and “CKD stages 3–5,” and clinical, biological, and immunological data as well as comorbidities and drugs affecting hemostasis at the time of biopsy. The histopathological data include the number of glomeruli, descriptive SNOMED codes assigned by the renal pathologist, and whether the diagnosis is based on light microscopy, immunofluorescence/immunohistochemistry, and/or electronic microscopy. Major complications are also collected as well as the final clinical diagnosis of nephropathy assigned by the senior nephrologist, using the ERA-PRD coding system, [33] as described above. The SRR-kidney biopsy also collects follow-up data (preferred time points at 1, 5, and 10 years), including immunosuppressive therapy and biological data. In 2019, the coverage was estimated to be 58% of all biopsies performed in adult native kidneys in the country.

#### SRR-dialysis

This was launched in 2002 in several regions and became a part of the national SRR in 2007. It collects data from patients on prevalent maintenance hemodialysis (HD) or peritoneal dialysis (PD). The patients are included if they are alive and, on any dialysis modality, between 15 September and 15 October. Each year, within this period, a mandatory yearly work-up occurring during a mid-week dialysis session is registered into SRR, and a large set of clinical and biological data, dialysis modality (conventional HD, hemodiafiltration (HDF), hemofiltration, home-HD, automated PD, continuous ambulatory PD), dialysis prescription and parameters, and selected medications are collected. Between two consecutive mandatory yearly data collection into SRR-dialysis, data from other mid-week dialysis sessions may be collected at the clinic's discretion. The coverage of participating dialysis clinics in Sweden is 100%, and 93%–99% of the prevalent dialysis population each year is estimated to be included.

#### SRR-access

This collects, at national level, detailed information on hemodialysis vascular accesses (central venous catheter, arteriovenous fistula, and graft) since 2011, and since 2017 registration of PD accesses has been integrated [35]. Beyond the type/subtype of dialysis access, the database includes the dates of access creation/insertion and its function, location, as well as complications and radiological/surgical interventions. Each event is recorded into the SRR-access enabling assessment of individual vascular access trajectories. External validation has demonstrated increasing coverage of registered vascular accesses between 2011 and 2017, reaching >80% after 2016 [35].

#### SRR-PROMs and QoL

These were introduced in the end of 2016, this SRR specific module enables the clinics to collect, via a digital application online or on paper format, patient-reported outcomes measures (PROMs), which encompass health-related QoL (HRQoL). HRQoL is assessed using the RAND-36 [36–38], a validated 36 item questionnaire that provides an eight-dimensional profile measured on a scale from 0 to 100–a higher score indicating a better perceived health status during the last 4 weeks. It assesses physical functioning, role limitations caused by physical problems (role physical), bodily pain, general health, vitality/energy/fatigue, social functioning, mental health/emotional well-being, and role limitations caused by mental health/emotional problems role emotional. An additional item refers to the patient's assessment of his/her health transition during the last 12 months. Other collected data include demographics, eGFR or KRT modality at the time of completion of the questionnaire. The response rate of RAND-36 is 42%–48% in dialysis patients taking part in the yearly cross-sectional evaluation.

#### SNR-special drug database 

This is the most recent module. It was implemented in 2018 with the aim to follow CKD patients treated with new therapies, where a closer follow-up is regarded valuable such as for tolvaptan, roxadustat, and difelikefalin—prescribed for specific medical conditions or delivered by a hospital pharmacist. It collects data on indication, date of start/discontinuation, dose and dose change, and selected adverse events. No formal external validation has been undertaken for this part of the registry yet.

### Data collection, data protection, and quality check

Data collection to SRR is implemented as a routine procedure at outpatient nephrology clinics, dialysis units, and transplantation centers. The main collected data are presented in Table 1 and include demographics, clinical and biological data, selected comorbidities, CKD-specific medications (except intravenous immunosuppressive medications), as well as scales evaluating different components of mental and physical health and HRQoL. Data are collected continuously—manually or automatically (Region Skåne)—through a secure web-based data electronic case report file, access to which requires dual authentication; thus, SRR is updated in real-time. The data entry is performed using drop-down lists and tool-tip information with definition and explanations. Laboratory data is entered as numerical digits, with pre-defined ranges and units to minimize data entry errors. Although biological parameters are analyzed at different laboratories across Sweden, all the laboratory departments are audited annually by the Swedish Government-run agency EQUALIS (Uppsala, Sweden, www.equalis.se/en) to ensure reproducibility and national standardization across healthcare systems. Data quality control checks are performed by the administrators continuously. Data is also validated systematically according to a pre-defined quality assurance program.

Confidentiality, security, and integrity of data are covered by OMDA Health Analytics information system as agreed with Region Jönköping, who is the general data controller of the SRR. All extracted data are pseudonymized by the SRR or the Swedish National Board of Health and Welfare, replacing the personal identification number with a random number. Data are encrypted and stored on a secure server in accordance with the European GDPR and the Swedish internal data security guidelines. The SRR data are regularly saved in highly secure servers.

The SRR data are presented annually in a yearly report (https://www.snronline.se), overall and stratified by clinic for selected quality control variables. The local clinics can access their own continuously updated reports and data using their site SRR login, for internal quality procedures. The quality control variables are also presented at the national website https://www.vardenisiffor.se, run by the Swedish Association of local Authorities and Regions.

### Follow-up

SRR includes both active (continuous follow-up by the reporting clinics) and passive (administrative cross-linkages with national healthcare registers) follow-up.

All patients are clinically evaluated at a frequency determined by the attending nephrologist, and SRR records continuously in real-time (SRR-epidemiology, SRR-CKD, SRR-access, SRR-biopsy) or annually (SRR-Dialysis, SRR-PROMs) each autumn one or more visits per patient. Each time the status of a patient changes, for example when transitioning from ND-CKD to KRT, or changing KRT modality (i.e. between dialysis modalities, from dialysis to kidney transplantation, and back to dialysis after kidney transplantation failure), the transition is documented in the SRR-epidemiology. The clinics also continuously update the registry with mortality data retrieved at the clinical follow-up, including the date and the cause of death, using the ERA classification system.

Passive follow-up is assessed annually when the SRR is cross-linked with information about vital status at the National Board of Health and Welfare to ensure completeness and no losses to follow-up. Patients are followed until death or emigration from the country. Thus, any patient who is ever registered in SRR will have follow-up for vital status and KRT initiation, regardless of whether they are referred from nephrology to another clinic or not.

### Linkage to national Government-run registries and other quality registries

**Figure 2:**
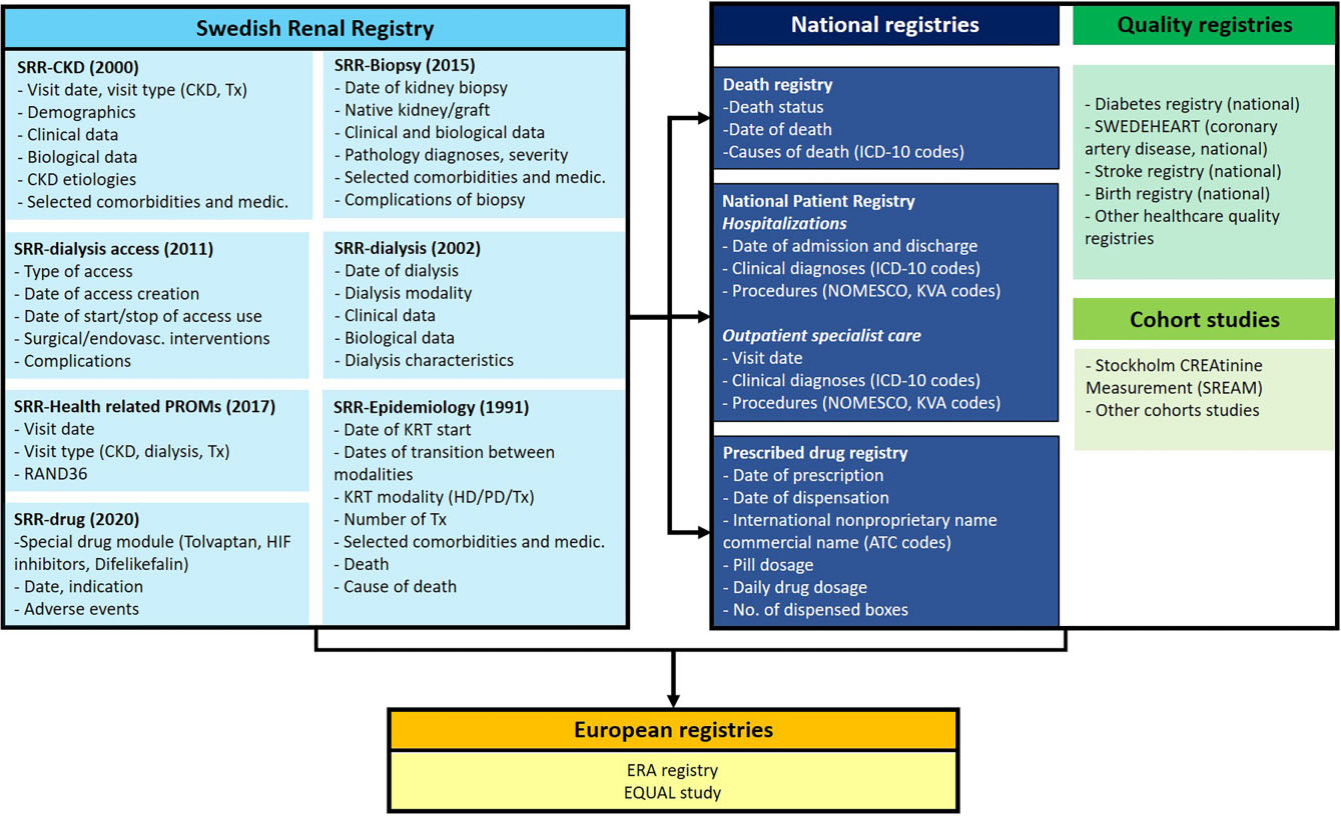
SRR databases and linkage.

Via each citizen's unique 10-digit personal identification number given at birth or at entry to the country as a legal immigrant, it is possible to link data from the SRR to several national Government-run registries for specified research projects [39, 40], such as the National Patient Registry, which provides information on clinical diagnoses and medical/surgical procedures performed in both inpatient and outpatient care in Swedish healthcare since 1997; the National Death Registry [39], which records information on the vital status with the date and the cause(s) of death; and the Prescribed Drug Registry [41], which provides information on issued and dispensed medications at any Swedish pharmacy, since 2005 (Fig. 2).

### SRR as a part of international CKD cohorts and research

The SRR-epidemiology part of the registry reports annually to the ERA Registry data on incident and prevalent patients on KRT, kidney transplantation, and mortality in Sweden [26]. The SRR-CKD part is also responsible for the Swedish data collection in the European QUALity (EQUAL)-study, a European prospective cohort study in elderly patients with CKD stage 4. In this prospective cohort study, ~300 patients in Sweden with an incident eGFR <20 ml/min/1.73 m^2^ are included and followed according to a specific protocol and written informed consent procedure [42]. The ERA Registry is responsible for the coordination of this study, which has included >1700 European patients.

### Statistical analyses

In the present article, we sought to describe the main information provided by each SRR database, without using any linked national registries, of incident and prevalent patients enrolled in SRR from 1 January 1991 to 31 December 2021, for the SRR-epidemiology. For other parts that were launched later, we present data spanning over the period 2008–2021, starting from when data were available for the different parts.

Baseline characteristics of patients are reported as frequencies and percentages for categorical variables and mean (SD) or median (Q1–Q3, first and third quartile) for continuous variables. CKD etiologies, vascular access at hemodialysis start, and QoL assessed using the RAND-36 questionnaire are presented graphically, by CKD status. Baseline characteristics of patients with KRT are also presented overall, and by blocks of five calendar years to explore trends over time in demographics, comorbidities, treatments, and KRT modalities. Incidence of KRT and KRT modalities are presented as percentages and per million population (pmp) values, using information on the total population of Sweden, by 31 December of each year (1991–2021), provided by Statistics Sweden, the official authority for population statistics in Sweden https://www.scb.se/.

## RESULTS

A total of 45 590 patients were included in the SRR-CKD, 4753 in the SRR-biopsy, 10 906 in the SRR-hemodialysis, 5128 in the SRR-PD, and 5490 in the SRR-QoL part.

### Baseline characteristics by CKD stage

Between 2008 and 2021, 45 590 new patients with ND-CKD [72 years, 16 656 (36%) women, eGFR 26 ml/min/1.73 m², uACR 28 mg/mmol] were enrolled in SRR (Table 2). Furthermore, there were 10 906 new patients on maintenance HD [68 years, 3798 (35%) women], and 5128 on PD [67 years, 1622 (32%) women] registered in the yearly cross-sectional survey. Between 2015 and 2021, information on kidney biopsies performed in 4753 unique patients [59 years, 1884 (40%) women, eGFR 37 ml/min/1.73 m², uACR 140 mg/mmol] were also collected.

**Table 2:** Baseline characteristics of patients registered in SRR (2008–2021), by CKD category.

	NA,	SRR-CKD	NA,	SRR-biopsy	NA,	HD^a^	NA,	PD^a^
	%	*N* = 45 590	%	*N* = 4 753	%	*N* = 10 906	%	*N* = 5 128
Clinical data								
age, years [Q1–Q3]	0.0	72.0 [63.0, 80.0]	0.0	59.2 [42.4, 70.8]	0.0	68.0 [56.0, 77.0]	0.0	67.0 [53.0, 76.0]
gender, women	0.0	16 656 (36.5)	0.0	1884 (39.6)	0.0	3798 (34.8)	0.0	1622 (31.6)
SBP, mmHg	7.6	138.9 (21.8)	0.9	134.2 (17.0)	1.4	143.1 (24.7)	10.5	136.1 (20.6)
DBP, mmHg	7.7	76.9 (11.9)	0.9	77.2 (10.5)	1.5	74.6 (15.4)	10.7	78.3 (12.2)
BMI, kg/m²	43.9	28.1 (5.9)	13.0	27.9 (6.4)	4.1	26.2 (5.8)	7.1	26.1 (4.7)
Comorbidities								
hypertension	3.6	41 114 (93.5)	2.8	3 244 (70.2)	0.5	10 382 (95.7)	0.2	5 054 (98.7)
diabetes mellitus	2.7	15 305 (34.5)	9.0	1 014 (23.4)	0.4	4161 (38.3)	0.8	1 787 (35.1)
ischemic heart disease	2.9	8 321 (18.8)	2.8	446 (9.7)	0.6	2 733 (25.2)	0.9	1 237 (24.3)
peripheral vascular disease	2.9	2 573 (5.8)	2.8	140 (3.0)	0.6	1 140 (10.5)	0.9	444 (8.7)
cerebrovascular disease	2.8	3 672 (8.3)	2.8	273 (5.9)	0.6	1 234 (11.4)	0.9	542 (10.7)
Laboratory data								
hemoglobin, g/dl	5.3	12.3 (1.7)	4.3	12.1 (2.1)	0.7	11.3 (1.4)	0.6	11.7 (1.4)
serum potassium, mmol/l	46.3	4.4 (0.5)			31.9	4.7 (0.7)	38.2	4.1 (0.6)
serum calcium, mmol/l	13.7	2.3 (0.2)			3.9	2.3 (0.2)	6.4	2.3 (0.2)
serum phosphorus, mmol/l	14.0	1.3 (0.3)			1.2	1.6 (0.5)	1.1	1.6 (0.4)
PTH, pmol/l	37.9	17.2 (18.6)			6.5	31.4 (35.2)	10.7	30.1 (28.7)
albumin, g/l	9.6	36.5 (5.0)	4.1	30.9 (8.3)	1.8	34.2 (5.3)	0.9	31.3 (5.5)
eGFR, ml/min per 1.73 m²	1.9	25 [19, 33]	2.9	37 [21, 62]				
eGFR stage	1.9		2.9					
>60		1 956 (4.4)		1 217 (26.4)				
45–59		2 299 (5.1)		605 (13.1)				
30–44		10 474 (23.4)		946 (20.5)				
15–29		23 415 (52.4)		1 143 (24.8)				
<15		6 573 (14.7)		703 (15.2)				
ACR, mg/mmol [Q1–Q3]	51.2	28 [5, 130]	14.5	142 [33, 362]				
ACR categories	51.2		14.5					
A1		3 849 (17.3)		215 (5.3)				
A2		7 504 (33.7)		734 (18.1)				
A3		10 886 (49.0)		3 114 (76.6)				
Medications								
RASi	5.5	24 653 (57.2)			1.6	4 282 (39.9)	0.6	2 384 (46.8)
diuretics	5.5	25 428 (59.0)			1.6	7 317 (68.2)	0.6	4 320 (84.8)
CCB	5.5	21 641 (50.2)			1.6	5 108 (47.6)	0.6	2 847 (55.9)
beta blockers	5.5	24 815 (57.6)			1.6	7 079 (65.9)	0.6	3 579 (70.2)
ESA	0.0	11 182 (24.5)			0.0	9 482 (86.9)	0.0	4 118 (80.3)
iron	5.5	5 934 (13.8)			1.6	7 469 (69.6)	0.6	1 619 (31.8)
phosphate binders	5.5	8 296 (19.3)			1.6	8 611 (80.2)	0.6	4 162 (81.7)
vitamin D	5.5	17 012 (39.5)			1.6	7 843 (73.1)	0.6	4 119 (80.8)
calcimimetic	5.5	502 (1.2)			1.6	1 253 (11.7)	0.6	631 (12.4)
lipid-lowering drugs	5.5	21 186 (49.2)			1.6	4 535 (42.2)	0.6	2 694 (52.9)

a Patients are included at their first registration in the dialysis cross-sectional survey taking place once a year, between 15 September and 15 October.

ACR, urinary albumine creatinine ratio; A1-A3, albuminuria level 1-3; BMI, body mass index; CCB, calcium channel blockers; DBP, diastolic blood pressure; ESA, erythropoeitin stimulating agents; N, number; NA, non available; SBP, systolic blood pressure; RASi, renin angiotensin system inhibitors; PTH, parathyroid hormone; Q, quartile.

**Figure 3:**
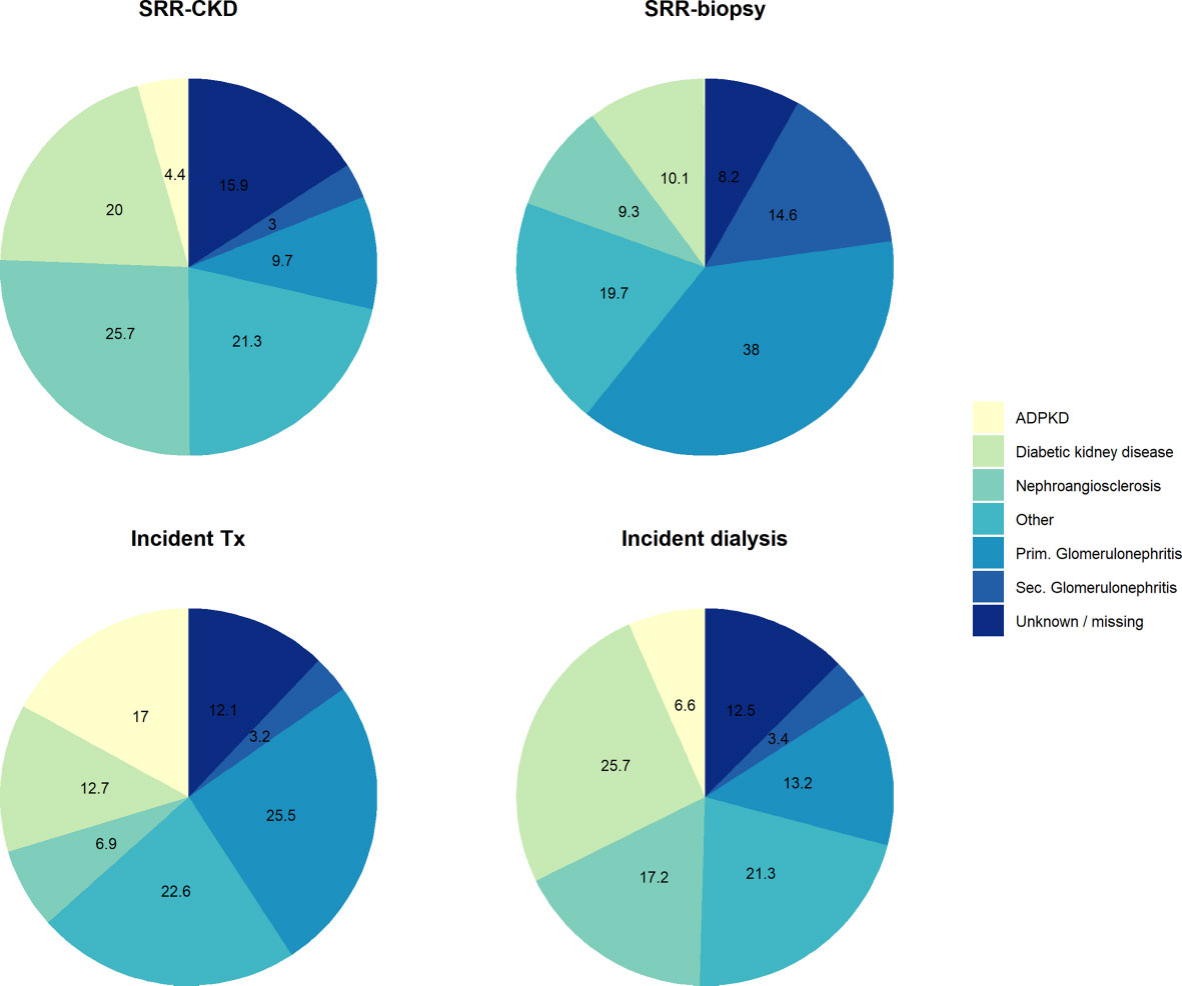
Etiologies of nephropathy (in percentage) classified according to the ERA coding system.

In general, CKD patients were more often men and had a high prevalence of cardiovascular risk factors and cardiovascular disease, which increased as CKD progressed (Table 2). Baseline characteristics were in general comparable between patients on HD and on PD. Diabetic kidney disease (19.8% and 25.6%) and nephroangiosclerosis (26.4% and 17.7%) were the most frequent nephropathies among new patients included in SRR-CKD and SRR-dialysis, whereas glomerulonephritis (52.6% and 28.7%) represented the main underlying CKD etiology in patients who underwent a kidney biopsy and in incident patients with kidney transplant (Fig. 3).

**Figure 4:**
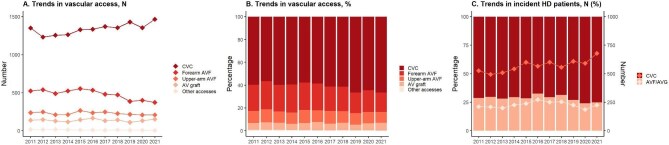
Temporal trends in vascular accesses for hemodialysis, 2011–2021. (**a**) and (**b**) The total number (a) and percentage (b) of placed vascular accesses, by type and calendar year. (**c**) Vascular accesses in incident patients on hemodialysis during the same period. AV, arteriovenous; AVF, arteriovenous fistula; AVG, arteriovenous graft; CVC, central venous catheter.

In new patients on HD (*N* = 10 906), conventional HD (69.5%) was more common than HDF (30.2%). Among the 5128 new patients on PD, 31% and 69% were on automated PD and continuous ambulatory PD, respectively. A total of 3917 unique PD catheters and 24 162 unique vascular accesses were registered in the SRR-access database. Overall, 61% of patients start HD on a catheter and 39% on arteriovenous fistula/graft or other accesses (Fig. 4). Among arteriovenous fistulas/arteriovenous grafts, endovascular interventions (*N* = 13 994) were more frequent than surgery (*N* = 4621).

**Figure 5:**
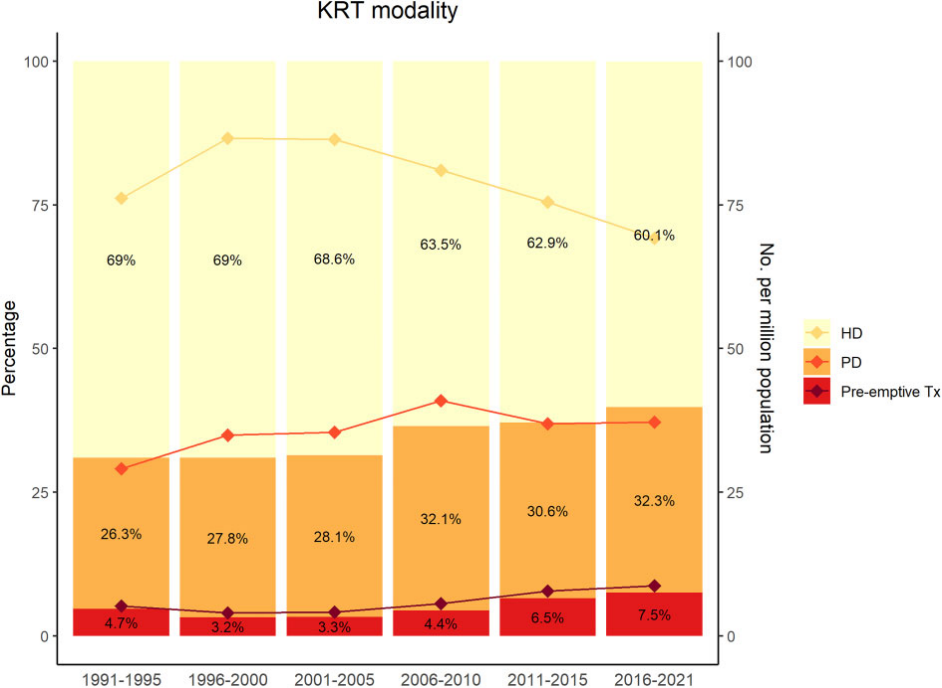
Trend over time of KRT modality.

Between 2016 and 2022, 5835 QoL questionnaires were filled in by 5490 patients (46% of the questionnaires in CKD, 30% in HD, 8% in PD, and 15% in kidney transplanted patients). Results indicate low scores in each of the eight dimensions as well as of the additional item health transition, with the lower scores in patients on dialysis than patients with ND-CKD, transplanted or not. Across CKD stages, physical dimensions were more affected than mental and social dimensions (Fig. 5).

### Trend over time in patients with kidney replacement therapy (1991-2021)

**Figure 6:**
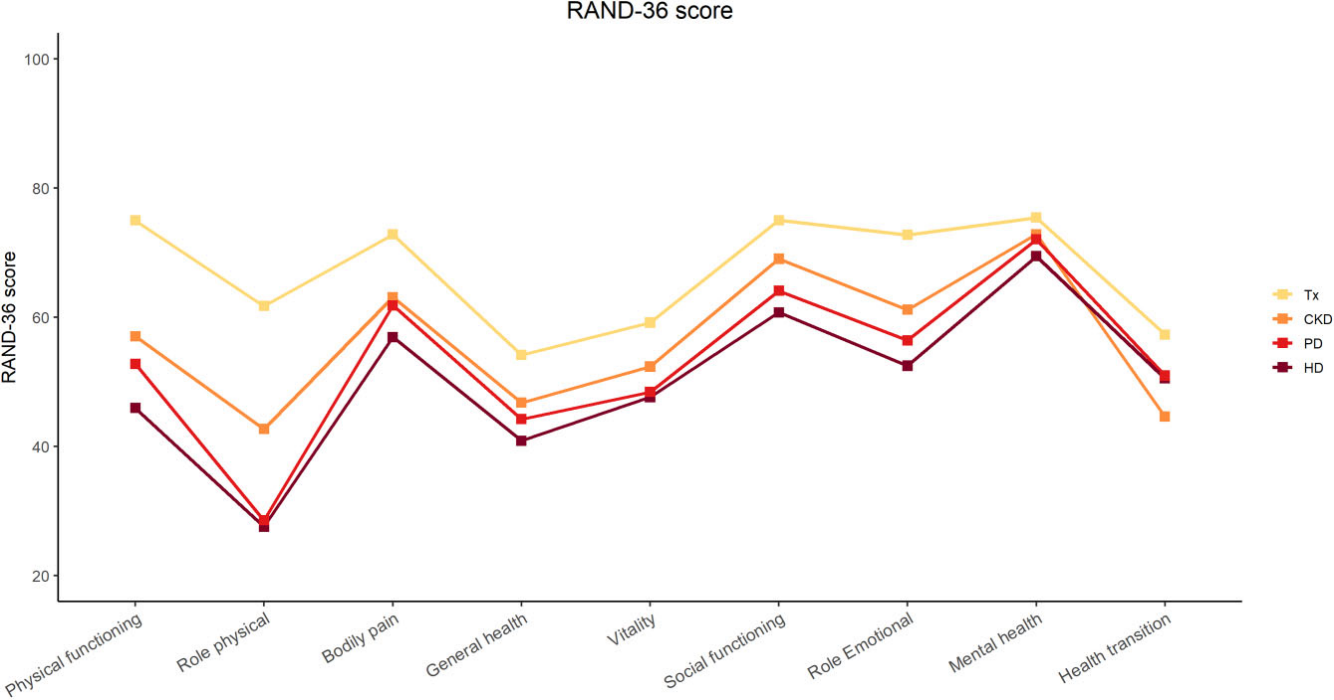
QoL and patient-reported outcomes, by eGFR category.

Between 1991 and 2021, the number of incident patients with KRT remained relatively stable, with approximately 1100 patients starting KRT each year in Sweden (Table 3). However, while the incidence of KRT was stable around 125 pmp from 2000 to 2010, the incidence dropped substantially during the two last 5-year block periods, reaching 115 pmp between 2016 and 2021. In parallel, the median age at KRT start slightly increased from 65 to 68 years with a higher proportion of men. The prevalence of diabetes and hypertension increased, while that of cardiovascular disease tended to decrease over time. At KRT start, the proportion of patients with diabetic nephropathy and nephroangiosclerosis increased over time, whereas that of glomerulonephritis decreased. The first choice of KRT modality also evolved over time, with a slight increase in PD (from 26.3% to 32.3%, *P* for trend <.01) while HD tended to decrease from 69% (86 pmp) to 60.1% (69 pmp), *P* < .01 (Table 3, Fig. 6). Between 1991 and 2021, 9668 kidney transplantations were performed in 8829 patients. The median age at the first kidney transplantation was 51 years (IQR 39–61) and 35% were women. Among the kidney transplant recipients, 5881 (67%) received a kidney from a deceased donor, while 2948 (33%) had a living donor transplantation. There were 1761 (5.1%) pre-emptive kidney transplantations over the entire period, with an increasing trend in both absolute (4.7% in 1991–1995 to 7.5% in 2016–2021, *P* < .01) and relative (5.2 pmp in 1991–1996 to 8.7 pmp in 2016–2021) terms (Table 3).

**Table 3:** Baseline characteristics of incident patients starting KRT (day), overall and by calendar year.

		Overall	1991–1995	1996–2000	2001–2005	2006–2010	2010–2015	2016–2021
	NA, %	*N* = 34 820	*N* = 4833	*N* = 5556	*N* = 5653	*N* = 5903	*N* = 5800	*N* = 7075
Demographics								
age, years [Q1–Q3]	0.0	67.0 [53.0, 76.0]	65.0 [50.0, 74.0]	67.0 [53.0, 75.0]	67.0 [54.0, 76.0]	67.0 [54.0, 76.0]	67.0 [53.0, 76.0]	68.0 [55.0, 76.0]
gender, women	0.0	12 131 (34.8)	1 735 (35.9)	2 056 (37.0)	1 977 (35.0)	2 036 (34.5)	1 960 (33.8)	2 367 (33.5)
comorbidities								
hypertension	0.3	20 423 (83.9)			4 526 (80.1)	4 856 (82.3)	47 96 (82.9)	6 245 (89.2)
diabetes mellitus	0.3	9 210 (37.9)			1 977 (35.0)	2 221 (37.6)	2 159 (37.3)	2 853 (40.9)
ischemic heart disease	0.4	6 950 (28.6)			1 845 (32.6)	1 858 (31.5)	1 561 (27.0)	1 686 (24.3)
peripheral vascular disease	0.5	2 849 (11.7)			749 (13.3)	739 (12.5)	657 (11.4)	704 (10.1)
cerebrovascular disease	0.4	2 888 (11.9)			685 (12.1)	713 (12.1)	672 (11.6)	818 (11.8)
Primary cause of kidney disease								
diabetic nephropathy		8 421 (24.2)	1 078 (22.3)	1 267 (22.8)	1 390 (24.6)	1 490 (25.2)	1 413 (24.4)	1 783 (25.2)
nephroangiosclerosis		4 682 (13.4)	371 (7.7)	588 (10.6)	652 (11.5)	742 (12.6)	909 (15.7)	1 420 (20.1)
primary glomerular disease		5 262 (15.1)	964 (19.9)	901 (16.2)	778 (13.8)	792 (13.4)	819 (14.1)	1 008 (14.2)
secondary glomerulonephritis		1 219 (3.5)	171 (3.5)	198 (3.6)	224 (4.0)	169 (2.9)	194 (3.3)	263 (3.7)
ADPKD		2 222 (6.4)	249 (5.2)	296 (5.3)	341 (6.0)	400 (6.8)	408 (7.0)	528 (7.5)
others		8 811 (25.3)	1 449 (30.0)	1 619 (29.1)	1 627 (28.8)	1 352 (22.9)	1 298 (22.4)	1 466 (20.7)
unknown		4 203 (12.1)	551 (11.4)	687 (12.4)	641 (11.3)	958 (16.2)	759 (13.1)	607 (8.6)
KRT modality^a^ (pmp)	0.0	**120.5**	**110.5**	**125.4**	**125.9**	**127.5**	**120.1**	**115.0**
HD		22 700 (65.2) 78.5	3 334 (69.0) 76.2	3 834 (69.0) 86.6	3 879 (68.6) 86.4	3 751 (63.5) 81.0	3 647 (62.9) 75.5	4 255 (60.1) 69.2
PD		10 359 (29.8) 35.8	1 272 (26.3) 29.1	1 544 (27.8) 34.9	1 588 (28.1) 35.4	1 892 (32.1) 40.9	1 776 (30.6) 36.9	2 287 (32.3) 37.2
pre-emptive transplantation		1 761 (5.1) 6.1	227 (4.7) 5.2	178 (3.2) 4.0	186 (3.3) 4.1	260 (4.4) 5.6	377 (6.5) 7.8	533 (7.5) 8.7

a The incidence of KRT at day 1, is presented as number, percentage, as well as pmp, overall, and stratified by KRT modality and calendar year.

## DISCUSSION

The nationwide SRR constitutes an important and powerful real-life research platform that includes well-phenotyped patients with CKD across all stages, referred to nephrologist care. The most original aspects of the SRR are first the ability to conduct translational research of both type 1, from bench to bedside and of type 2, from bedside to population; and second in the SRR, the patients are followed across their different stages of severity and transitions, also incorporating results from kidney biopsies and dialysis accesses among the collected data. The presentation of this nationwide registry illustrates important challenges for improving clinical management and outcomes in CKD. Patients with CKD referred to nephrologist care have a very high-risk profile, characterized by old age, a high burden of comorbidities—especially cardiovascular—polypharmacy, and a poor QoL, which steadily increases as CKD progresses. There is also a substantial percentage of dialysis access complications in dialysis patients.

### Comparison with other European CKD registries

There are several other CKD registries in Europe. The ERA Registry is the largest, collecting data on KRT in up to 35 different countries including Sweden. [26] Age, sex, and primary cause of kidney disease in patients from the SRR-epidemiology part align well with the characteristics of patients from other European KRT cohorts, as presented in the last ERA Registry Annual reports [26].

The number of registries/cohorts collecting data on CKD-ND is lower. In 2021, a review summarized them and included registry/cohorts from the Czech Republic, French-speaking Belgium, Finland, France, Norway, Romania, and Sweden [43]. The characteristics of the SRR-CKD non-dialysis population are not readily comparable to other CKD registries in Europe because of differences in inclusion criteria, because many of the registries have not published annual reports, or have a low coverage as they did not include patients from the whole country. However, there are also several well-defined prospective cohort studies collecting data in patients with CKD-ND. The most well-known of these cohorts are the CKD Outcomes and Practice Patterns Study (CKDopps) cohort study [44], the CKD-Renal Epidemiology and Information Network (CKD-REIN) cohort, and the German CKD (G-CKD) cohort. In comparison to the patients included in CKD-REIN and G-CKD, patients enrolled in SRR are older, but with a similar proportion of men versus women [8, 45]. While the patients from the G-CKD cohort had substantially higher eGFR at baseline, the patients in CKD-REIN had only slightly higher eGFR. Patients in SRR-CKD generally have higher levels of albuminuria. These differences in characteristics are probably because SRR-CKD composes an unselected referred CKD population targeting more severe CKD stages, and patients recruited in clinical cohorts generally are healthier than those not participating [46].

### Trend over time of patients reaching KRT

In Sweden, over the past two decades, the annual number of patients starting dialysis remains relatively stable with decreasing incidence rates pmp over time, despite an aging population. Similar trends have been observed for many of the western European countries [47]. Our study shows that the incidence of KRT in Sweden is lower than the European mean, and three times lower than that of the USA [48]. The reasons behind the large variation in KRT incidence across the world were recently described in an analysis of the DOPPS data. It includes differences in the prevalence of CKD risk factors such as diabetes, but also uptake of nephroprotective treatments, and selection in characteristics of patients starting KRT [49]. Between 1991 and 2021, we observed interesting changes in the trend of pre-emptive kidney transplantation, with an incidence increasing from 5.2 to 8.7 pmp. A similar trend was also observed in other European countries, indicating a generally increased awareness of the advantages of pre-emptive kidney transplantation [50].

Over the follow-up period, we observe an increase in the prevalence of diabetes and hypertension in Sweden that, interestingly, translates into a higher percentage of patients starting KRT due to diabetic nephropathy or nephroangiosclerosis, while in the meantime, the prevalence of cardiovascular disease—including ischemic heart disease, peripheral artery disease, and cerebrovascular disease—tends to decrease. By contrast, the percentage of patients starting KRT with a glomerulonephritis as the primary renal disease tends to decrease over time, probably thanks to improved clinical management and major therapeutic advances over the last decades. These results could also be mirrored by previous studies in SRR, demonstrating improving mortality and a lower risk of major cardiovascular events in patients on HD over time [51].

### The roles of SRR in healthcare quality assessment and research

The main objective of the registry is to ensure the adherence to clinical guidelines and recommended treatments in all regions of Sweden. The self-evaluation for each clinic is facilitated by pre-designed reports for a set of indicators of quality of care, which are easily followed to help improve nephrology care. The indicators are also presented to stakeholders and are sometimes used locally to allocate resources and highlight processes in need of improvement. At local, regional, and national levels, the results from SRR are used to follow the implementation of new guidelines, for instance, the KDIGO guidelines on anemia [52], or clinical practices [53, 54]. In Sweden, healthcare quality registers are regarded as an important part of the evaluation of treatment practices according to the national system for “knowledge-driven management” in healthcare.

However, another important aspect of SRR is its role in research. The collection of a wide range of prospective data, along with the unique possibility to link to many other national healthcare registries, allows the investigation of a many different exposures (especially risk factors) including clinical, biological, and medical conditions, but also a large set of outcomes and clinical practices in patients with CKD from all etiologies, in almost all CKD stages (non-dialysis CKD, kidney transplant, and maintenance dialysis periods), and also allows for comparisons at the international level, to improve patient care and outcomes. An important aspect, which makes SRR different from many other registries, is the possibility to follow individual patient trajectories, transitioning from one CKD stage to another (e.g. non-dialysis CKD stage 5 to dialysis or kidney transplantation), to assess changes within KRT modality, and to study patients not starting KRT, i.e. opting for conservative care. The SRR has contributed to >200 scientific publications since it was first created on a wide variety of research topics and clinical questions: for example, studies on when (in terms of eGFR) to initiate dialysis [55], pharmacoepidemiologic studies [56, 57], descriptive epidemiology [58, 59], clinical nephrology [60–62], validation of prediction models [63], vascular access [64], patient-reported outcomes [65, 66], and health-economic analyses [67].

### Strengths and limitations

Strengths include a national representative cohort of patients with CKD, from the early stages to kidney failure and transplantation, an extensive data collection, a long-term follow-up with virtually no loss to follow-up, and the setting of the Swedish universal tax-funded healthcare that minimizes selection bias from disparate access to healthcare. The data linkages to other national healthcare sources further ensures the complete collection data of high quality with no loss to follow-up, for example on socioeconomics, clinical diagnoses, dispensed medications, vital status, and cause(s) of death.

The SRR has some limitations. First, although the SRR encourages inclusion of patients with all CKD stages, patients are not necessarily enrolled at the time of diagnosis, but mainly when they reach eGFR <45–30 ml/min per 1.73 m², as historically decided by each participating site, depending on the number of patients and allocated resources. This has been overcome since the launch of SRR-biopsy in 2015, where patients are included regardless of eGFR at the time of kidney biopsy. Second, information on urine sediment, smoking/alcohol consumption, physical activity, dietary pattern (except prescribed low protein diet), and health behaviors are lacking, and histological features have only become available in the recent years. Third, laboratory tests are not collected using a standardized protocol or centrally measured using standardized procedures, which may lead to intra- and inter-individual differences. Nevertheless, all laboratory departments are annually audited in Sweden to ensure national standardization across healthcare systems, and the adherence to reporting guidelines (for example frequency of follow-up visits) are centrally reviewed by the SRR administrative validation procedure. Moreover, non-mandatory laboratory tests could be measured (but not registered) or not. Fourth, the SRR does not collect any blood, urine, or tissue samples for research purposes. Finally, SRR predominantly includes patients of white ethnicity, thus, extrapolation of the findings to other population should be done with caution. However, results of SRR studies may be seen as an opportunity to complement those from other multi-ethnic cohorts such as those from the USA.

## CONCLUSION

The SRR provides a large nationwide “real-life platform” of patients with CKD, in which it is possible to investigate a wide range of exposures, outcomes, and clinical practices at national and international levels. SRR aims to address gaps in our understanding of CKD by answering key research questions about the etiology, prognosis, clinical management, long-term outcomes, HRQoL, clinical practices, and healthcare utilization in CKD and beyond, as well as to test novel mechanistic hypotheses, with the aim to improve clinical management and to reduce the burden of CKD.

## Data Availability

The data underlying this article cannot be shared publicly due to the privacy of individuals that participated in the study. The data may be shared on reasonable request for academic research collaborations that fulfill GDPR as well as national and institutional ethics regulations and standards by contacting Dr Marie Evans (marie.evans@ki.se).
